# Reconsidering the best minimally invasive approach for patients with herlyn-werner-wunderlich syndrome: Should we push the frontiers for a better outcome?

**DOI:** 10.3389/fsurg.2023.1158753

**Published:** 2023-04-11

**Authors:** Graziella Moufawad, Amal Ayed, Zaki Sleiman

**Affiliations:** ^1^ Department of Obstetrics and Gynecology, Lebanese American University Medical Center- Rizk Hospital, Beirut, Lebanon; ^2^ Department of Obstetrics and Gynecology, Farwaniah Hospital, Kuwait, Kuwait

**Keywords:** OHVIRA syndrome, laparoscopy, hysterectomy, management, fertility, obstetrical outcomes

## Abstract

Obstructed hemivagina and ipsilateral renal agenesis (OHVIRA) syndrome is a rare congenital defect of the Mullerian ducts characterized by uterine didelphys, unilateral obstructed hemivagina, and ipsilateral renal agenesis. It frequently presents during puberty, with complications such as pelvic pain, pelvic inflammatory disease and infertility. Surgical management is the mainstay treatment. A vaginal access for septum resection is usually used. However, it can be in difficult in several situations such as a very proximal septum with a small bulge, or in the case of virgin patients with social considerations regarding the hymenal ring integrity. Thus, a laparoscopic approach may be a beneficial alternative. In particular, laparoscopic hemi hysterectomy has recently gained remarkable interest due to its added benefit of treating the cause rather than treating only the symptoms. It removes the source of the bleeding, thus stopping the flow. However, it transforms a bicornuate uterus into a unicornuate uterus, leading to some obstetrical concerns. Should we push the frontiers further and consider laparoscopic hemi hysterectomy for better outcomes as the mainstay management of patients with OHVIRA syndrome?

## Introduction

Obstructed hemivagina and ipsilateral renal agenesis (OHVIRA) syndrome, previously known as “Herlyn-Werner-Wunderlich Syndrome” is a rare congenital defect of the Mullerian ducts, classified as **U3b C2 V2** upon the European Society of Human Reproduction and Embryology ESHRE classification (1).

It is characterized by a triad of uterine didelphys, unilateral obstructed hemivagina, and ipsilateral renal agenesis. Patients with OHVIRA syndrome present most commonly during puberty with pain, infertility, or a complicated obstetrical history (2). Incidence varies between 0.1 to 3.8% (3). This syndrome was believed to be due a multifactorial etiology. However, evidence supports an embryological cause. An abnormal development of the female genital tract, namely the Mullerian and Wolfian ducts is behind the development of OHVIRA syndrome (3).

The mainstay management of OHVIRA syndrome is surgical, with the surgeon's expertise being an important factor in the success of the treatment. The treatment of these patients relies either on the dissection of the septum or in some cases a hemi hysterectomy for the obstructed horn.

The objective of this review is to highlight the advantages of a laparoscopy over the pure vaginal route. Should we continue to rely on this approach as a minimally invasive surgery in the treatment of OHVIRA syndrome for better outcomes and an improved management plan?

### OHVIRA syndrome and associated symptoms

OHVIRA syndrome usually presents early on during the pubertal period. Diagnosis usually occurs after menarche due to retrograde menstrual flow which occurs in obstructive symptoms. As a result, endometriosis, pelvic inflammatory disease and infertility are serious complications which follow (4). In a systematic review of 734 cases performed by Kudela et al., to study the multiple variants of OHVIRA syndrome, isolated hematocolpos or hydrocolpos was found in 55.9% of the cases (3). Similarly, endometriosis was found in 13.6% of the cases which underwent laparoscopy or laparotomy; however, this could be an underestimation since not all patients underwent the latter 2 procedures (3). In another study conducted by Santos et al., it was concluded that retrograde flow in OHVIRA syndrome can lead to endometriosis in 23% of the cases (5).

Mullerian duct anomalies in general have been highly associated with gynecological and infertility symptoms, in particular: primary amenorrhea, dysmenorrhea, endometriosis, infertility and preterm labor (6). A report was published by Freytag et al. discussing uterine anomalies and endometriosis. It was concluded that endometriosis and congenital uterine anomalies have been frequently reported to coexist together in the literature (7). Endometriosis is associated with obstructive anomalies of the uterus, and hence, it can be concluded that OHVIRA syndrome can lead to secondary endometriosis.

### Treatment options

Early detection and surgical treatment is necessary as it relieves symptoms and prevents complications such as infertility and endometriosis (8). Treatment of Mullerian anomalies is aimed at treating the anatomy of the defect (5). Surgical management with vaginal septum resection was once considered the ultimate surgical approach. However, this vaginal approach is faced with several obstacles which has led to it being reconsidered. As already mentioned, OHVIRA syndrome usually presents during pubertal periods, a time when most of the patients have not yet had sexual intercourse. Thus, this vaginal approach is not always feasible especially in virgin patients. Hymen integrity is of utmost importance in certain societies such as Arab and Middle Eastern countries. Thus, surgical procedures requiring a vaginal access in patients with an intact hymen might be a source of societal contraindication or discomfort (9).

**Figure 1:**
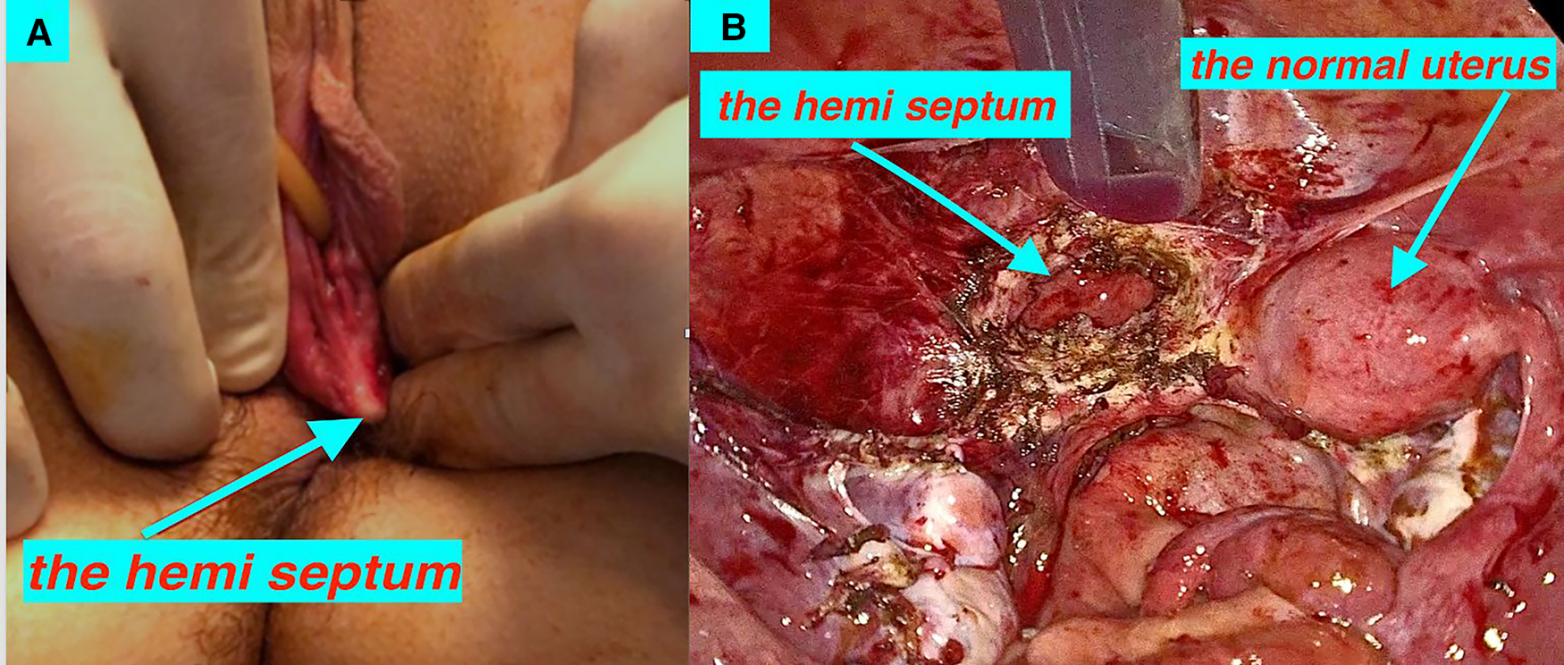
The laparoscopic grasper pushing downward the high septum.

Apart from cases of virginity, other cases ensued which necessitated an abdominal approach to assist a vaginal approach in septum resection. Dissection of the septum can be a pure vaginal or a hysteroscopic approach but with the advancement of minimally invasive surgery, laparoscopy started gaining its interest in the management of such cases. This holds especially true in cases where a small hematocolpos is present, rendering a pure vaginal approach difficult as it is unable to well define the landmark for septum resection. In 2019, a novel laparoscopic technique was described to assist the vaginal approach in a 16 year old woman with OHVIRA syndrome who had an intact hymenal ring and refused any vaginal procedure (10). During the laparoscopy, a posterior 1 cm colpotomy was performed, the hematocolpos was drained, a grasper was introduced thus pushing the high septum downwards through the vagina providing an easier, safer, and more accessible landmark for the dissection (Figure 1A). Similarly in 2020, Boyraz et al. reported the case of a 30 year old woman with OHVIRA syndrome who had an intact hymen. A transverse anterior colpotomy was performed, in this case *via* laparoscopy, followed by vaginal septum resection and suturing of the vaginal incision (11). In addition to accurately defining the landmark of septum resection in case of a small hematocolpos, the presence of hematosalpinx, and endometriosis necessitates a laparoscopic approach for a better surgical management.

## Discussion

1. The importance of laparoscopy:

The vaginal hemi septum is usually highly situated and the hematocolpos can be either small or big in size depending on the distensibility of the vaginal wall. This makes the access to the septum through a vaginal approach quite challenging carrying a risk of injury to the nearby bladder and rectum. Thus, the use of laparoscopy to assist vaginal access is of great importance, especially when a small bulge is the case. Laparoscopic assistance helps the surgeon better visualize the septum and the exact landmark for better dissection, avoiding nearby organs and unnecessary incisions. According to Yakistiran et al., laparoscopy is considered the gold standard for diagnosis with an additional therapeutic benefit over other techniques, including drainage of hematometra or hematocolpos, resection of the vaginal septum which is our main concern in case of OHVIRA syndrome, or marsupialization (12). Similarly, Sleiman et al. described the use of laparoscopy in the management of OHVIRA syndrome with a high vaginal septum, in order to avoid laparotomy. In this case, complications of laparotomy, which include infection, increased blood loss, and post operative pain are avoided. An additional benefit over vaginal access is allowing a better definition of the surgical landmark for a safer septum resection (10).

The dissection of the vaginal septum will release the pain, but in most of the cases a peritoneal implication due to the retrograde flow is coexistent. It consists of a hematosalpinx, tubo ovarian adhesions, and in some cases, secondary endometriosis. All these complications are conditions that could highly impact the fertility of patients. Thus, in order to treat this aspect of the pathology, hysteroscopy and vaginal surgery are never enough. Concomitant laparoscopy can assist not only allowing a safer vaginal dissection of the septum but also in performing a salpingectomy, adhesiolysis, or excision of endometriomas whenever present (13).

2. Laparoscopic hemihysterectomy:

Laparoscopic hemi hysterectomy can be an alternative for septum dissection in the case of reclosure of the septum or in the case of virgin patients who, for social considerations, totally refuse a vaginal approach. Figure 1B shows the laparoscopic view of the hemiseptum after hemihysterectomy without closure of the colopotomy. According to Huseyin et al., laparoscopic hemi hysterectomy is the preferred management approach in several cases, especially in cases of proximal vaginal septum resulting in an impossible vaginoplasty (13). This management plan is of high importance in cases with a delayed diagnosis, complicated by pyocolpos, pyometra, and pyosalpinx from perforations in the septum (13). A study published in 2020 by Gungor et al. reported that in cases of increased distance between the perineum and the hematocolpos, hemihysterectomy was successfully performed instead of vaginoplasty (14). This further highlights the importance of hemihystrectomy in complicated cases of OHVIRA with a proximal vaginal septum and a delayed diagnosis. Huseyin et al. described the case of a complicated OHVIRA syndrome patient with late diagnosis. A vaginal access was not enough to drain the pyometra and a distorted anatomy with extensive pelvic adhesions were encountered. A conversion into laparoscopic hemihysterectomy was then successfully performed. They thus concluded that when performed by experienced laparoscopic surgeons, laparoscopic hemi hysterectomy is safe and feasible in the management of OHVIRA syndrome despite the presence of extensive pelvic adhesions (13). Furthermore, laparoscopic hemi hysterectomy can be performed at ease and with improved safety due to the absence of the ureter on the side ipsilateral to the obstruction.

### Do we push the frontiers further?

After discussing the safety, feasibility, and advantages of laparoscopic hemi hysterectomy, should we push the frontiers of our surgery and propose this alternative as a gold standard treatment? The advantage is that we are treating the cause, not only the symptoms. A laparoscopic hysterectomy treats the source of the flow instead of only releasing the obstruction. However, we are creating a new problem in the fact that we are transforming a patient with a bicornuate uterus into a patient with a unicornuate uterus. Here lies the question of fertility, pregnancy rates and obstetric complications. Does this really jeopardize the fertility and obstetrical outcome of the patient?

A systematic review was conducted by Y.Y Chan et al. to study the effect of congenital uterine anomalies on reproductive outcomes. It was concluded that unification defects, i.e., unicornuate and bicornuate uteri are not associated with decreased fertility compared to normal uteri, but they are both associated with miscarriage and preterm delivery. Statistical analysis revealed that bicornuate uteri are more commonly associated with first trimester miscarriage rates (3.40 vs. 2.25, *p*-value < 0.05) compared to unicornuate uteri. However, bicornuate uteri have a lower risk of preterm labor when compared to unicornuate uteri (2.55 vs. 3.47, *p*-value < 0.001) (15). Thus, transforming a patient with a bicornuate uterus to a patient with a unicornuate uterus leads to higher risk of preterm labor, but to lesser first trimester miscarriage rates, with a similar fertility rates.

In a study conducted by Golan et al. to study the obstetric outcomes in women with congenital uterine anomalies, patients with unicornuate uteri had a 61% chance of delivering at term, and only a 7% chance of early abortions. Thus patients with unicornate uteri were considered to have good obstetrical outcomes when compared with patients with a T shaped uterus which had a 47% rate of early term abortions and only 21% chance of delivering at term (16).

We can presume then that before the surgical correction, there exists the problem of a unicornuate uterus together with the presence peritoneal adhesions that can block the functional tube due to retrograde flow. After surgical correction, and in cases of septum dissection uniquely, the bicornuate uterus will still exist. However, with hemi hysterectomy a unicornuate uterus will arise with its similar fertility rates but lower first trimester miscarriage rates. This further strengthens the importance of hemihysterectomy in terms of ameliorating obstetrical and fertility outcomes, in addition to treating the symptoms and removing their causes.

There exist reports of delivery in patients with OHVIRA syndrome treated with hemihysterectomy. Cappello et al. reported the case of a 28 year old woman with OHVIRA syndrome who was treated during her adolescence with laparoscopic hemihysterectomy. Her pregnancy lead to a delivery at a term of 34 weeks GA by cesarean section due to breech presentation. Thus, pregnancies at after hemihysterectomy are possible and can lead to delivery around the late preterm period.

Evidence is still lacking regarding pregnancy rates in patients with OHVIRA syndrome before septum resection and pregnancy rates occuring in those patients in the normal horn post vaginal septum resection (17). The systematic review published by Y. Y Chan et al., which included 9 studies comprising 3,805 women, has proved that unicornuate uteri behave similarly to bicornuate uteri in terms of fertility rates.

## Conclusion

Based upon the above mentioned evidence based facts, we can push the frontiers further and consider that a pure laparoscopic approach with hemi hysterectomy is, when needed, a feasible and beneficial alternative approach in the management of obstructive hemivagina with ipsilateral renal agenesis. This approach has better outcomes in terms of symptomatic treatment and pain relief. In addition, it allows avoiding a vaginal access and thus shortcutting the obstacle of an intact hymen when virginity is required by the patient and her family. Finally, this approach is important in terms of treating the peritoneal implications of the retrograde flow that can have a remarkable effect on the tubal function of the normal uterus.
